# Precision Medicine for Alzheimer’s Disease Prevention

**DOI:** 10.3390/healthcare6030082

**Published:** 2018-07-13

**Authors:** Cara L. Berkowitz, Lisa Mosconi, Olivia Scheyer, Aneela Rahman, Hollie Hristov, Richard S. Isaacson

**Affiliations:** Department of Neurology, Weill Cornell Medicine, New York, NY 10021, USA; cab2040@med.cornell.edu (C.L.B.); lim2035@med.cornell.edu (L.M.); ols2011@med.cornell.edu (O.S.); anr2781@med.cornell.edu (A.R.); how2005@med.cornell.edu (H.H.)

**Keywords:** Alzheimer’s disease prevention, precision medicine, clinical precision medicine, apolipoprotein ε4, APOE, methylenetetrahydrofolate reductase, MTHFR

## Abstract

Precision medicine is an approach to medical treatment and prevention that takes into account individual variability in genes, environment, and lifestyle and allows for personalization that is based on factors that may affect the response to treatment. Several genetic and epigenetic risk factors have been shown to increase susceptibility to late-onset Alzheimer’s disease (AD). As such, it may be beneficial to integrate genetic risk factors into the AD prevention approach, which in the past has primarily been focused on universal risk-reduction strategies for the general population rather than individualized interventions in a targeted fashion. This review discusses examples of a “one-size-fits-all” versus clinical precision medicine AD prevention strategy, in which the precision medicine approach considers two genes that can be commercially sequenced for polymorphisms associated with AD, apolipoprotein E (APOE), and methylenetetrahydrofolate reductase (MTHFR). Comparing these two distinct approaches provides support for a clinical precision medicine prevention strategy, which may ultimately lead to more favorable patient outcomes as the interventions are targeted to address individualized risks.

## 1. Introduction to Precision Medicine

The National Institute of Health (NIH), along with several other research centers, has created the Precision Medicine Initiative as a new way of approaching medicine with a targeted and patient-centered focus [1]. Specifically, they have defined precision medicine as an “emerging approach for disease treatment and prevention that takes into account individual variability in genes, environment, and lifestyle for each person” [1]. This approach to the practice of medicine has a high potential for treating the nuances of individuals with different genetics, lifestyle factors, and medical comorbidities that may affect their response to treatment. Since its initiation, many fields, including oncology [2] and cardiology [3], have begun refocusing their efforts to more precision-based approaches to practicing medicine. The role that genetics plays in the development of late-onset Alzheimer’s disease (AD) has been widely studied, with one study estimating genetics to account for more than 50% of the phenotypic variance [4]. However, the field of AD prevention has yet to fully advance intervention strategies from universal risk-reduction approaches to targeted interventions based on personalized risk factors, including genetics. In the following discussion, we review examples of a universal “one-size-fits-all” prevention strategy without any distinction that is based on genetics or other personalized risk factors versus a clinical precision medicine approach. From a practical clinical perspective, we have focused on two genes that can be commercially sequenced for polymorphisms that are associated with AD and that physicians may order to help better inform patient care. These include the most well-characterized genetic influencer on late-onset AD risk, apolipoprotein E (APOE), and another potential genetic influencer, methylenetetrahydrofolate reductase (MTHFR). With the increasing ease of both clinical lab-based, as well as direct-to-consumer genetic sequencing, a precision medicine approach that incorporates established genetic factors may be feasible and may also favorably affect patient outcomes by addressing individualized risks as well as pharmacogenomics and nutrigenomic considerations for AD.

## 2. “One-Size-Fits-All” Approach to AD Prevention

Randomized studies in AD prevention have traditionally used either single or multiple interventions to determine efficacy across a host of clinical outcome measures (e.g., cognitive function, serum biomarkers, brain imaging). The vast majority of these studies have used a “one-size-fits-all” approach to targeting diet, exercise, and other lifestyle factors without accounting for any individual genetic variables. Two large-scale randomized control trials (RCTs), the Multidomain Alzheimer Prevention Trial (MAPT) and Prevention of Dementia by Intensive Vascular Care (PreDIVA) trial did not show improvements in cognitive functioning with lifestyle interventions, including nutrition, physical activity, cognitive engagement, and management of comorbidities [5,6,7]. However, these studies used populations that were already experiencing some degree of cognitive decline or dementia. As AD starts developing in the brain decades before clinical symptoms become apparent [8], these study populations may not have been optimized to benefit from lifestyle modifications since individuals experiencing cognitive decline may already be beyond a critical window for AD prevention [9]. 

The Finnish Geriatric Intervention Study to Prevent Cognitive Impairment and Disability (FINGER) was the first multicenter RCT to investigate the effects of similar lifestyle interventions on cognitive functioning in non-impaired individuals at risk for cognitive decline [10]. The results of the FINGER trial demonstrated that all individuals, regardless of baseline cognition, cardiovascular risk, demographics, or socioeconomic status improved with lifestyle interventions [11,12]. While not included in the initial study, a sub-analysis of the FINGER trial further explored the impact of a particular genetic factor, APOE, on lifestyle interventions in this cohort. This sub-analysis is further discussed in the ‘Precision Medicine Approach to AD Prevention’ section.

Several other prevention studies have shown improvement in cognitive function by implementing universal lifestyle interventions in non-impaired individuals, but with highly variable results. The two categories of interventions with the most robust evidence thus far include nutrition (including dietary patterns and single or multi-nutrients) and physical exercise. The Mediterranean diet is one example of a dietary pattern that has been extensively studied for AD prevention. A recent meta-analysis investigating the impact of the Mediterranean diet on cognitive functioning showed that there was a lower risk of cognitive decline and conversion to mild cognitive impairment (MCI) or AD in subjects with higher adherence to the diet [13]. However, there are also studies that have failed to demonstrate benefits of diet-specific interventions for AD prevention as well as various studies with mixed findings about which particular dietary interventions are the most beneficial [14,15,16].

Another well-studied dietary intervention focusing on single or multi-nutrients in the area of AD prevention has aimed to optimize levels of omega-3 polyunsaturated fatty acids (n-3 PUFA), most specifically, docosahexaenoic acid (DHA) [17,18,19,20,21,22,23]. Epidemiological evidence indicates that regular fish consumption and higher n-3 PUFA levels may reduce the risk for age-associated cognitive decline and AD [24]. Also, higher blood n-3 PUFA levels are protective of cortical structures [25], and chronic fish oil supplementation is associated with increased posterior cingulate activation in non-demented older adults [26]. Additional studies have shown improvement in cognitive function or decreased risk of AD in healthy individuals with DHA supplementation [18,19,20] or with consumption of fish high in omega-3s once per week [20]. However, other studies have suggested that there is no benefit of omega-3 supplementation with regard to cognitive function and AD prevention [22,23].

Another lifestyle intervention that has been studied for AD prevention with variable results is physical activity. A meta-analysis on the role of physical activity in AD prevention concluded that physical activity significantly decreased the risk of developing AD [27]. In addition, an RCT that looked at the impact of physical activity on cognitive functioning demonstrated that individuals who participated in six months of physical activity showed improvement in cognitive functioning up to 18 months later [28]. Researchers have also investigated the timing and intensity of physical activity. One study found that light and vigorous activity in mid-life and light and moderate activity in late-life were associated with lower risks of developing MCI [29]. However, similar to the variations in the nutrition data, there have been varied findings and conclusions about the type and intensity of physical activity that are most effective at reducing the risk of AD, as well as studies demonstrating no risk reduction from physical activity [30,31,32].

The discrepancy in the data on nutritional interventions and physical activity may be related to a lack of a “one-size-fits-all” solution, and modifications in diet, exercise, and other lifestyle factors may need to be personalized to have maximum efficacy. Unlike universal risk reduction approaches, precision medicine strategies allow for incorporation of individual risk factors that may uniquely affect the response to interventions.

## 3. Precision Medicine Approach to AD Prevention 

A precision medicine approach to AD prevention will need to fully utilize the genome in order to make personalized recommendations. In this section, we discuss some of the genetic influencers on late-onset AD that can currently be ordered by a practicing physician and provide examples of a targeted precision medicine approach based on these genetic factors. 

## 4. APOE and AD Prevention

One of the most well-established genetic influencers on late-onset AD risk is APOE [33], which codes for the apolipoprotein E protein [34]. There are three major polymorphisms at the APOE loci: APOE ε2, ε3, and ε4. Studies have shown that APOE genotype significantly impacts the risk of AD. Specifically, the ε4 allele has been associated with an increased risk of AD [35], while the ε2 allele has been associated with a decreased risk [36]. In addition, the risk of developing AD is even greater in individuals with two copies of the ε4 allele when compared to those with only one copy [37].

Several pathophysiologic mechanisms may explain why APOE ε4 is associated with an increased risk of AD and APOE ε2 is associated with a decreased risk. First, the three major alleles code for proteins with different molecular properties that result in different binding properties of apolipoprotein E to β-amyloid. This difference in binding may contribute to the enhanced accumulation of β-amyloid plaques that was observed in ε4 individuals, which is one of the pathologic markers of AD [38]. Furthermore, their distinct molecular properties also result in differences in their ability to bind to and transport lipids. Studies have demonstrated that there are allele-specific interactions of APOE with both LDL and HDL receptors that play an important role in the development of atherosclerosis, which is one of the major risk factors for AD [34]. As the ε4 allele has been estimated to account for 27.3% of late-onset AD risk (with a heritability of 80%) and with emerging evidence that potential risk-reduction interventions may be preferentially effective (or less effective) depending on presence of the ε4 allele, it may be important to incorporate this genotype into the AD prevention approach [39].

There are several AD prevention interventions that can be personalized based on APOE genotype. Although the FINGER trial showed no significant differences in cognitive function between APOE genotypes with their multimodal lifestyle interventions, a within-group analysis of the APOE ε4 allele demonstrated that there was a significant difference in certain treatment versus control scores only for individuals with ε4 alleles [12]. This suggests that some inherent difference exists between individuals with and without APOE ε4 alleles that impacted the effectiveness of the interventions. Therefore, additional trials with larger sample sizes and more statistical power are important in order to discern the impact of APOE on these multimodal interventions.

Other single-factor studies have demonstrated that AD prevention interventions can be targeted based on APOE genotype. A systematic review of studies that altered dietary fat composition showed that changes in total cholesterol, LDL, and HDL were most significant in individuals with APOE ε4 alleles in 15 of the studies [40]. In another study, researchers found that, in response to a Mediterranean diet, both individuals with and without APOE ε4 alleles showed improvements in cognitive functioning, as measured by the Mini Mental State Exam (MMSE), but only individuals without ε4 alleles showed improvement in the clock drawing test, a measure of executive functioning and spatial reasoning [41]. Tailoring strategies to APOE genotype can also be effective for physical activity interventions. For example, one study demonstrated that sedentary individuals with ε4 alleles were at greater risk of developing MCI, whereas physically active individuals without ε4 alleles were at decreased risk [29]. Another study demonstrated that aerobic fitness was correlated with higher cognitive performance in ε4 homozygotes [42]. Similarly, with regard to omega-3 fatty acids, three recent RCTs showed an improvement in cognitive function with DHA supplementation in non-impaired individuals with ε4 alleles [43].

While a comprehensive review of the literature is beyond the scope of this manuscript, these studies demonstrate that, based on APOE genotype, individuals may exhibit more significant responses to different lifestyle interventions. For example, individuals with ε4 alleles may experience greater changes in total cholesterol, LDL, and HDL in response to reductions in dietary fat, whereas individuals without an ε4 allele might show greater improvement in certain cognitive functions from the Mediterranean diet. In addition, physical activity may benefit all individuals but may have increased efficacy for those with ε4 alleles. Similarly, DHA supplementation may also lead to greater improvement in cognitive function in those with at least one ε4 allele. Overall, genotype-specific strategies such as these may benefit patients by using an evidence-based approach and utilizing specific targeted interventions that were shown to be the most effective for individuals with their same genotype. Additional research to further elucidate the role of APOE genotype on different dietary, physical activity, and other lifestyle interventions will be important in the future as the precision medicine approach to AD prevention continues to develop.

## 5. MTHFR and AD Prevention

The MTHFR gene, which codes for the methylenetetrahydrofolate reductase protein, is another potential genetic contributor to AD and is also readily available for physicians to order in commercial labs. Several MTHFR polymorphisms have been described in the literature [44], but two polymorphisms, C677T and A1298C, have had the greatest investigation as to their association with AD [45]. These polymorphisms also appear to have a high prevalence in the general population [46], and one study reported that 92.5% of its AD subjects had at least one of these MTHFR polymorphisms [45].

The association between MTHFR polymorphisms and AD may relate to the catalytic role that the MTHFR protein plays as the rate-limiting step in the conversion of homocysteine into methionine, with the B-vitamins folate and cobalamin serving as cofactors [47]. Homocysteine is an amino acid that is involved in inflammation and has been associated with cognitive decline and an increased risk of AD [48,49]. One study in cognitively healthy individuals found that baseline homocysteine levels inversely correlated with cognitive testing scores and rates of cognitive decline over a five-year period [48]. Similarly, another study of 1000 individuals from the Framingham cohort looked at non-impaired individuals at baseline and showed a strong positive correlation between baseline homocysteine and the risk of dementia up to 11 years later [49]. Another longitudinal study showed there was an 88% increased rate of cognitive decline over ten years associated with doubling the homocysteine level from 10 mg/L to 20 mg/L [50].

Changes in the MTHFR protein that alter its catalytic function, such as seen in the C677T and A1298C polymorphisms, result in higher levels of serum homocysteine [51], and therefore, have the potential to increase the risk of AD. Several studies have shown an association between the A1298C polymorphism and an increased risk of AD [52], but not with the C677T polymorphism [52,53]. However, another study showed that the combination of these two polymorphisms with a third A1793G polymorphism, together known as Haplotype C, was associated with a decreased risk of AD [54]. Therefore, further research into the relationship between these polymorphisms and the risk of AD is warranted.

Similar to APOE, MTHFR genotype status may allow for targeted AD prevention interventions. B-vitamin supplementation (cyanocobalamin, folic acid, and B6) has been shown to slow cognitive decline in individuals with elevated homocysteine levels [55,56]. Several trials have studied a combination of B vitamins to determine whether lowering homocysteine can impact cognitive function and/or brain pathology [55]. While there is limited evidence thus far, individuals with one or more MTHFR polymorphisms may potentially benefit from genotype-specific recommendations. For example, as individuals with certain MTHFR polymorphisms have decreased catalytic ability of the MTHFR protein, replacing the traditional B-vitamins with their methylated counterparts (methylcobalamin for cyanocobalamin and methyltetrahydrofolate [5-MTH] for folic acid) that do not require hepatic conversion to active forms may increase the outcomes. One study demonstrated that 5-MTH supplementation in individuals with C677T and A1298C polymorphisms significantly increased the serum folate concentration when compared to folic acid, but it did not result in differences in the serum homocysteine concentration [57]. Additional studies evaluating the impact of methylated B-vitamins for specific MTHFR polymorphisms and AD risk may therefore help to advance the field of precision medicine for AD prevention. 

## 6. Other Genetic Influencers on AD Prevention

In addition to the discussed polymorphisms in the APOE and MTHFR genes, recent genome-wide association studies (GWAS) have identified several other single nucleotide polymorphisms (SNPs) that are associated with an increased risk of AD: CLU, CR1, and PICALM. Although these genes are not yet routinely available for sequencing commercially, the impact of polymorphisms at these loci on dietary interventions for AD prevention has recently been investigated [58,59]. One study demonstrated that improvements in cognitive function in response to the Mediterranean diet differed depending on which polymorphisms an individual had [41]. These findings provide further evidence that genetics may modify the effectiveness of AD prevention interventions. As this trial only investigated the impact of the Mediterranean diet on polymorphisms at these loci, other dietary interventions as well as other lifestyle interventions should be explored in a similar manner. In addition, there are many other known genetic risk factors for AD, such as TOMM40, which have yet to be explored regarding their impact on lifestyle interventions for AD prevention [60]. However, these genes are also not yet routinely commercially available for sequencing. A discussion of all of the genes that are involved in AD risk is beyond the scope of this paper, but it is discussed further in an Alzgene meta-analysis [61].

## 7. Conclusions and Future Directions

This review considered examples of two approaches to AD prevention: a universal “one-size-fits-all” approach, which uses generalized prevention strategies for all individuals, and a clinical precision medicine approach, which factors in genotype-specific intervention strategies. While both approaches have merit, utilizing a precision medicine approach offers the opportunity to personalize interventions that are based on factors that may impact the efficacy of the interventions. Genotype-specific intervention strategies, in particular, hold a great deal of promise for advancing the field of AD prevention toward more personalized and effective intervention strategies.

Investigation into the impact of different genetic factors on AD prevention will continue to become more practicable through online genetic repositories that are available to the scientific community. For example, the Alzheimer’s Disease Sequencing Project (ADSP) and Alzheimer’s Disease Neuroimaging Initiative (ADNI) are ongoing large-scale whole-exome and whole-genome sequencing projects in individuals with AD available through the NIA Genetics of Alzheimer’s Disease Data Storage Site (NIAGADS) and the database of Genotypes and Phenotypes (dbGaP) [62]. This increased availability of genetic data will provide additional resources to investigate the impact of various genetic factors on AD prevention interventions in the future. 

In addition, there has been an exponential growth in the ability of consumers to order personal genomic testing on their own via a number of commercially available testing kits. In the United States, the Food and Drug Administration approved the first direct-to-consumer tests that provide genetic risk information for a subset of medical conditions, including APOE [63]. Further, despite these commercial tests not being meant for clinical purposes, it has also become more common for patients (and even some physicians) to use a number of online tools to further investigate the raw data provided by these tests. Websites such as Promethiase.com and Snpedia.com may be utilized, although there are currently no professional guidelines and/or standards on how to do this [64].

We should also be mindful of the ethical implications of integrating genetic risk factors into clinical practice. While the Risk Evaluation and Education for Alzheimer’s Disease (REVEAL) study demonstrated that APOE ε4 disclosure to adult children of AD patients did not result in significant short-term psychological effects, the long-term effects have not been evaluated [65,66]. Clinicians should weigh the potential risks and benefits of disclosing genetic risk factors to their patients and should counsel patients accordingly prior to disclosing genotype status [67]. Referral to a certified genetic counselor should also be considered when clinically indicated. Over the last five years at the Alzheimer’s Prevention Clinic (APC) at Weill Cornell Medicine and NewYork-Presbyterian, the majority of patients (over 95%) have consented to receive APOE and MTHFR testing [68]. Counseling is initially provided in person by either of the two treating clinicians (a board-certified Neurologist or Family Nurse Practitioner). Patients are also asked to complete an online course via AlzU.org that explains genetic risk for AD and limitations of these tests [69]. In select cases, when patients have additional questions or concerns about testing, patients may be referred to a genetic counselor. In all patients with a family history that is highly suggestive of early-onset (autosomal dominant) AD, patients are referred to a genetic counselor prior to any genetic testing. Studies are ongoing to determine whether APOE and MTHFR polymorphism disclosure to APC patients impacts outcomes (e.g., compliance with recommendations, psychological measures including anxiety and depression). Furthermore, additional analyses are planned to determine whether clinical outcomes (e.g., cognitive performance, blood biomarkers of AD risk) are differentially impacted by APOE and MTHFR genotype. Generally speaking, the use of genetic testing as a part of clinical evaluation and patient care has been a favorable addition in the opinion of the treating clinicians, although further study is warranted in a broader subset of clinicians and in diverse patient cohorts.

Finally, it is important to consider the limitations of a genetic-based precision medicine approach to AD prevention. The genomic-centered foundation that forms the core of precision medicine reduces diseases to their molecular and cellular processes. However, there are many risk factors for AD in which the exact pathogenesis is not fully understood. A precision medicine approach that relies solely on genetics may miss some of the underlying mechanisms that are important for AD prevention but as of yet are not fully established. In addition to genetics, there are many other important aspects of a precision medicine approach to AD prevention, including medical comorbidities such as hypertension [70,71], diabetes [72], and hyperlipidemia [73,74], which have been associated with an increased risk of developing AD. There are also other lifestyle factors in addition to diet, exercise, and omega-3 fatty acids, such as smoking status, alcohol consumption, and cognitive engagement, which may play a role in AD prevention. Therefore, a precision medicine approach should also encompass recommendations to target these lifestyle factors and medical comorbidities on an individual basis. All of these factors need to be considered together to maximize a precision medicine approach that targets AD prevention strategies to the individual. Ultimately, genetics should be incorporated as one part of an overarching precision medicine approach to individualize AD prevention strategies.
